# Chikungunya Infection in India: Results of a Prospective Hospital Based Multi-Centric Study

**DOI:** 10.1371/journal.pone.0030025

**Published:** 2012-02-17

**Authors:** Pratima Ray, Vinod H. Ratagiri, Sushil K. Kabra, Rakesh Lodha, Sumit Sharma, B. S. Sharma, Mani Kalaivani, Naveet Wig

**Affiliations:** 1 Department of Pediatrics, All India Institute of Medical Sciences, New Delhi, India; 2 Department of Medicine, All India Institute of Medical Sciences, New Delhi, India; 3 Department of Biostatistics, All India Institute of Medical Sciences, New Delhi, India; 4 Department of Pediatrics, Karnataka Institute of Medical Sciences, Hubli, Karnataka, India; 5 Department of Pediatrics, Sawai Man Singh Medical College, Jaipur, Rajasthan, India; Emory University School of Medicine, United States of America

## Abstract

**Background:**

Chikungunya (CHIKV) has recently seen a re-emergence in India with high morbidity. However, the epidemiology and disease burden remain largely undetermined. A prospective multi-centric study was conducted to evaluate clinical, epidemiological and virological features of chikugunya infection in patients with acute febrile illness from various geographical regions of India.

**Methods and Findings:**

A total of 540 patients with fever of up to 7days duration were enrolled at Karnataka Institute of Medical Sciences (KIMS), Karnataka (South); Sawai Man Singh Medical College (SMS) Rajasthan (West), and All India Institute of Medical Sciences (AIIMS) New Delhi (North) from June 2008 to May 2009. Serum specimens were screened for chikungunya infection concurrently through RT-PCR and serology (IgM). Phylogenetic analysis was performed using Bioedit and Mega2 programs. Chikungunya infection was detected in 25.37% patients by RT-PCR and/or IgM-ELISA. Highest cases were detected in south (49.36%) followed by west (16.28%) and north (0.56%) India. A difference in proportion of positives by RT-PCR/ELISA with regard to duration of fever was observed (p<0.05). Rashes, joint pain/swelling, abdominal pain and vomiting was frequently observed among chikungunya confirmed cases (p<0.05). Adults were affected more than children. Anti-CHIK antibodies (IgM) were detected for more than 60days of fever onset. Phylogenetic analysis based on E1 gene from KIMS patients (n = 15) revealed ∼99% homology clustering with Central/East African genotype. An amino acid change from lysine to glutamine at position 132 of E1 gene was frequently observed among strains infecting children.

**Conclusions:**

The study documented re-emergence of chikungunya in high frequencies and severe morbidity in south and west India but rare in north. The study emphasizes the need for continuous surveillance for disease burden using multiple diagnostic tests and also warrants the need for an appropriate molecular diagnostic for early detection of chikungunya virus.

## Introduction

Chikungunya virus (CHIKV) is an enveloped positive-strand RNA virus belonging to genus *Alphavirus* of the family *Togaviridae* [1], [2]. It is an epidemic viral disease responsible for significant global public health problem mainly in Asian and African continents [3]–[9]. Efforts are underway in developing prevention strategy. Recently a VLP based chikungunya vaccine was developed in USA that was found to be protective in primates [10]. Chikungunya infection is generally characterized by fever and joint pains with additional symptoms including chills, vomiting, nausea, headache and rashes [11]–[13].

Historical data report the first detection of chikungunya in 1952 in the Makonde Plateau in Africa [14]–[15] where the virus is known to be maintained in the sylvatic cycle of wild primates and mosquitoes such as *Aedes taylori* [16]–[17]. Later in 1958 it was detected in urban Asia such as Thailand mainly transmitted by *Aedes aegypti* [18]–[20]. In India, where both *Aedes aegypti* and *Aedes albopictus* are known to exist and are widely prevalent during the post monsoon season, CHIKV was first detected in 1963 in West Bengal [18]. It was followed by several epidemics in Chennai, Pondicherry, Vellore, Visakhapatnam, Rajmundry, Kakinada, Nagpur and Barsi between 1964 and 1973 [21]. Recently CHIKV resurfaced in India affecting several South Indian states [21], [22]. The outbreak started in 2005 from the coastal regions of Andhra Pradesh and Karnataka [6], [23]. With more than 1.3 million people estimated to be affected CHIKV prevailed across 150 districts of 8 states in India [23]. Despite the number estimated, the actual disease burden was thought to be much higher due to potential underestimation from lack of accurate reporting [24].

CHIKV being an RNA virus is susceptible to high mutation rates which may help the virus to evade the immune response and thus adapt efficiently. However, phylogenetic analysis of E1 gene of CHIKV indicates only three lineages with distinct genotypic and antigenic characteristics i.e. the “Central/East African genotype”, the “Asian genotype” and the “West African genotype” [25]. CHIKV strains with an Asian genotype of E1 gene were reportedly detected during the 1963–73 outbreaks in India, while the more recent outbreaks since 2005 have been caused by the “Central/East African genotype” [21]. Additionally, a mutation at 226 amino-acid (Valine–Alanine) of E1 gene was observed during the recent outbreaks and has been associated with the more efficient replication of CHIKV in *Aedes albopictus* [26].

The high morbidity and loss in daily activity associated with CHIKV infection results in considerable economic loss among the affected nations, specifically India [27]. This emphasizes the need to have a detailed understanding of epidemiology and strain diversity for planning a prevention strategy. Towards this end the present study aimed to evaluate the disease prevalence at various geographical regions and assess the genomic diversity among CHIKV strains infecting the Indian population.

## Methods

### Study sites

Patients were recruited at three distantly located regions of India (North, West and South) from 1^st^ June, 2008 through 31^st^ May, 2009. Patient enrolment was carried out year round at Karnataka Institute of Medical Sciences (KIMS), Hubli, Karnataka (south); Sawai Man Singh Medical College (SMS) Jaipur, Rajasthan (West), and All India Institute of Medical Sciences (AIIMS) New Delhi (north) with AIIMS as the coordinating and testing centre.

### Patients and clinical specimens

Patient enrolment involved those with fever ≤7 days duration at the time of their visit to the Out/Inpatient departments at AIIMS (n = 178), KIMS (n = 233) and SMS (n = 129) hospitals and those who were willing to participate in the study (n = 540). The study was approved by each Institution's Ethics Committee (Institutional Ethics Committee, AIIMS; Ethics Committee SMS Medical College, Ethics Committee Karnataka Institute of medical Sciences, Hubli). All patients (or for children parents or guardians) gave written informed consents. Demographic details, clinical data including symptoms and onset and duration of fever were recorded in the specified proforma. Approximately 3 ml of blood was collected from each patient enrolled at the time of admission. Convalescent phase (15–90 days of fever onset) sample could be collected from 30 patients only at KIMS center. Sera were separated from the blood by centrifugation and stored at −70°C in aliquots till further use [28]. Specimens from KIMS and SMS centers were subsequently transferred to AIIMS center within two to three months of collection. Both RTPCR assay and serology (IgM) were employed to test CHIKV in all study specimens at the AIIMS centre.

### Diagnostic RT-PCR

Genomic viral RNA from CHIKV infected culture-lysate/patient sera were extracted using QIA Amp Viral RNA minikit (QIAGEN, Germany) according to manufacturer's protocol. The 294b E1 gene fragment of CHIKV was amplified with Qiagen one Step RT-PCR kit as described previously [29]. Briefly, the RNA fragment was reverse transcribed and amplified in a single step and the 294 bp amplified product was analyzed on 2% agarose gel as reported earlier [29].

### IgM-ELISA

All serum specimens were screened for CHIKV specific IgM antibodies by ELISA using a commercial kit (Standard Diagnostics, Inc., Korea) according to manufacturer's recommended procedure.

### Nucleotide sequencing

Sequences were determined from the 913 bp E1-amplicon (nt 10246–11158) that had been generated following RT-PCR (n = 15) with forward (5′-TACCCATTCATGTGGGGC-3′) and reverse (5′-TGACTATGTGGTCCTTCGGGGG-3′) primers. Amplification product was gel purified by the Gene-Clean purification method (Q-biogene, Cambridge, UK) and sequenced using a previously described method [30]. Lineage identification and assessment of evolutionary relationships were performed using Bioedit and Mega2 programs as described previously [30].

### Statistical analysis

Statistical analysis was performed using Stata statistical software, version 7.0 (StataCorp LP, College Station, TX, USA).

## Results

### Clinical features among suspected chikungunya patients

Table 1 lists patient demographics from a total of 540 cases with fever up to 7days duration regardless of chikungunya virus infection. Among the clinical features recorded, joint pain (62.8%) and headache (63.3%) were most frequently observed while other features included abdominal pain (48.1%), vomiting (43.9%), rash (36.1%), joint swelling (27.8%), cough (27.2%) and restlessness (21.3%). Symptoms such as diarrhea, rhinitis, conjunctival congestion, hepatomegaly, splenomegaly and lymphadenopathy were rarely observed (Table 1). Symptoms commonly caused by CHIKV i.e. joint pain, joint swelling, abdominal pain and conjuctival congestions were more common among patients at KIMS as compared to SMS and AIIMS.

**Table 1:** Clinical features observed among patients (n = 540) screened for CHIKV across various regions of India during 2008–2009.

Age, Sex &Clinical features	Delhi (n = 178)	Karnataka (n = 233)	Rajasthan (n = 129)	Total (n = 540)
Mean Age ± SD (in years)	10.51±8.16	25.75±19.88	8.38±3.83	16.61±16.15
Male	121(68.0)	114(48.9)	94(72.9)	329(60.9)
Rash	20(11.2)	110(47.2)	65(50.4)	195(36.1)
Erythematous	14(7.9)	101(43.3)	50(38.8)	165(30.6)
Maculopapular	12(6.7)	12(5.1)	13(10.1)	37(6.8)
Headache	111(62.4)	179(76.8)	52(40.3)	342(63.3)
Joint pain	81(45.5)	208(89.3)	50(38.8)	339(62.8)
Joint swelling	7(3.9)	119(51.1)	24(18.6)	150(27.8)
Abdominal Pain	65(36.5)	147(63.1)	48(37.2)	260(48.1)
Vomiting	61(34.3)	126(54.1)	50(38.8)	237(43.9)
Diarrhea	13(7.3)	11(4.7)	15(11.6)	39(7.2)
Rhinitis*	20(13.7)	26(25.2)	23(17.8)	69(18.2)
Cough	57(32.0)	50(21.5)	40(31.0)	147(27.2)
Restlessness	9(5.1)	78(33.5)	28(21.7)	115(21.3)
Hess test	22(12.4)	0(0.0)	0(0.0)	22(4.1)
Conjunctival congestion	5(2.8)	42(18.0)	3(2.3)	50(9.3)
Hepatomegaly	4(2.2)	31(13.3)	5(3.9)	40(7.4)
Splenomegaly	3(1.7)	10(4.3)	1(0.8)	14(2.6)

Note. Data are no. (%) of patients.

* Rhinitis was recorded for only 378 cases of which 146,103 and 129 at Delhi, Karnataka and Rajasthan respectively.

### Laboratory based diagnostics of Chikungunya viruses

CHIKV infection was tested both for the presence of anti-CHIKV IgM antibodies by ELISA and CHIKV RNA by RT-PCR. A total of 137 (25.4%) subjects tested positive for CHIKV through either of the two tests. A solitary case was detected at AIIMS while relatively higher percentages were observed at SMS (16.3%) and KIMS (49.4%) (Table 2). Rate of chikungunya detection was nearly similar between ELISA and RT-PCR (Table 2). Year round detection of chikungunya was observed at KIMS while at SMS it was rarely detected during summer. Moreover, majority of the CHIKV cases were observed during monsoon and post monsoon seasons both at SMS and KIMS.

**Table 2:** Serological and genomic based detection of chikungunya infection among 540 clinically suspected cases.

		No. (%) of positivity		
Study center	No. of subjects	IgM ELISA	RT-PCR (E1 gene)	Total positivity
**Delhi**	178	1 (0.56)	**-**	1 (0.56)
**Karnataka**	233	52 (22.32)	64 (27.47)	115* (49.36)
**Rajasthan**	129	16 (12.40)	5 (3.88)	21 (16.28)
**All**	540	69 (12.78)	69 (12.78)	137* (25.37)

*One sample was positive by both IgM serology & CHIKV E1 RT-PCR.

### Time kinetics of CHIKV RNA detection by RT-PCR and anti-CHIKV IgM antibodies by ELISA

**Figure 1:**
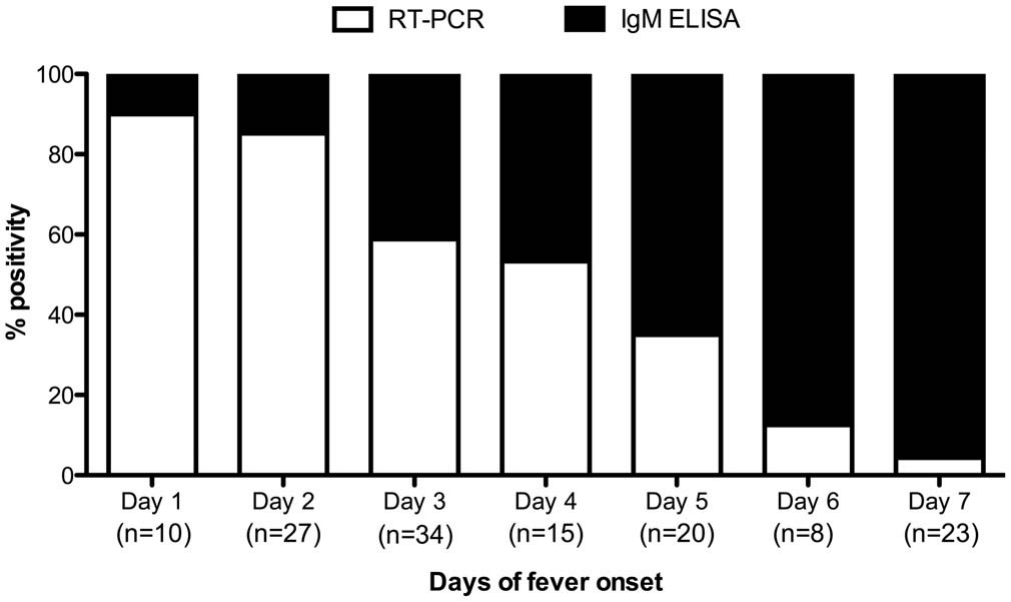
Proportion of CHIKV-positivity by RT-PCR or IgM ELISA according to days of fever onset. n denotes the total number of CHIKV positive cases.

Chikungunya detection was performed by both RT-PCR and IgM-ELISA with detection rates ranging from 4.3%–90% for RT-PCR and 10%–95.6% for ELISA depending on the days of fever onset (Figure 1). This difference in detection rates between RT-PCR and ELISA with respect to days of fever onset was found to be statistically significant through chi square analysis (p<0.05). RT-PCR assay was more sensitive till first 4 days of fever onset declining after day 4 at which point serology (IgM) was observed to be more effective (Figure 1).

Additionally 30 cases from KIMS were assessed for persistence of IgM during convalescence (15–90 days of fever onset). While majority of the chikungunya positive cases (9 of 11) were detected anti-CHIK IgM between 15–90 days post illness, none of the chikungunya negative patients (n = 19) were detected the same (data not shown).

### Association of clinical features with CHIKV laboratory diagnosis

Comparison of clinical features between chikungunya positive and negative patients demonstrated rashes, headache, joint pains, joint swelling, abdominal pain, cough and vomiting to be significantly associated with CHIKV confirmed cases (p<0.05) whereas differences for other symptoms were not found significant (Table 3).

**Table 3:** Comparison of clinical symptoms between CHIKV-positive subjects and CHIKV-negative subjects.

Clinical features(Number of patients having the clinical features)	No (%) Positive by EitherRT-PCR or IgM-ELISA (n = 137)	No (%) Negative by both assay (n = 403)	P value^a^
Rashes (n = 195)	68(49.6)	127(31.5)	0.0002
Headache (n = 342)	104 (75.9)	238 (59.1)	0.0006
Joint pain (n = 339)	117 (85.4)	222 (55.1)	<0.0001
Joint swelling (n = 150)	69 (50.4)	81 (20.1)	<0.0001
Abdominal pain (n = 260)	86 (62.8)	174 (43.2)	0.0001
Diarrhea (n = 39)	5 (3.6)	34 (8.4)	>0.05
Cough (n = 147)	24 (17.5)	123 (30.5)	0.0045
Vomiting (n = 237)	71 (51.8)	166 (41.2)	0.0387

Note. Data are no. (%) of patients.

a : p value was calculated using Chi square test for association of clinical symptoms between CHIKV positive and CHIKV negative cases.

### Age wise distribution of CHIKV infection among the study population

**Figure 2:**
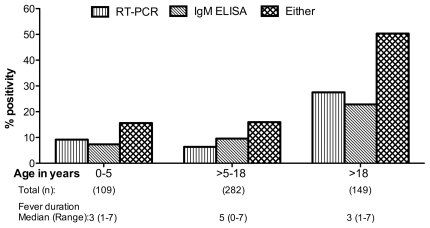
Age distribution of chikungunya patients at hospitals in Karnataka and Rajasthan, June 2008 through May 2009.

We compared the rate of CHIKV prevalence based on patient age groups 0–5, >5–18 and >18 years of age and observed significantly higher detection (p<0.05) among adult populations (aged >18 years). An apparent increase in frequency of CHIKV detection with age, from about 16% cases among children to 50% among adults was observed (Figure 2).

In addition Chi square analysis revealed significant differences for presence of rashes, headache, joint pain and joint swelling (p<0.05), between children (<18 years) and adults (>18 years) (Table 4). Interestingly, while most of the rashes among adult cases (>18 years) were of erythrematous type, that of children were maculaopapular type (<18 years) (data not shown).

**Table 4:** Comparison of clinical symptoms among chikungunya positive patients of different age groups.

Symptoms	Age groups			p value*	Odds Ratio (95% CI)*
	0–5 years (n = 17)	>5–18 years (n = 45)	>18 years (n = 75)		
Rash	5 (29.41%)	15 (33.33%)	48 (64%)	0.0002	3.73(1.83–7.60)
Headache	6 (35.29%)	26 (57.78%)	72 (96%)	<0.0001	22.5 (6.4–79.14)
Joint Pain	11 (64.71%)	32 (71.11%)	74 (98.67%)	<0.0001	32.7 (4.23–252.9)
Joint Swelling	4 (23.53%)	10 (22.22%)	55 (73.33%)	<0.0001	9.43 (4.3–20.67)
Abdominal Pain	10 (58.82%)	27 (60%)	49 (65.33%)	>0.05	1.27 (0.63–2.55)
Vomiting	8 (47.06%)	27 (60%)	36 (48%)	>0.05	0.71 (0.36–1.4)

**Note**. Data are no. (%) of patients.

*P value and Odds ratio was calculated using chi square test between patients of >18 years and up to 18 years of age.

### Phylogenetic tree based on E1 gene of CHIKV demonstrates high homology with strains belonging to Central/East African genotype

**Figure 3:**
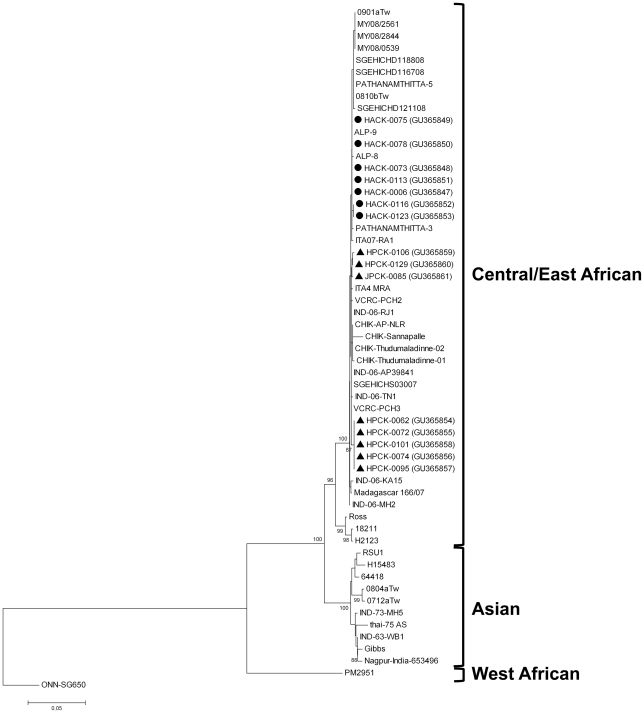
Phylogenetic analysis of the E1 gene (nt 10246 to 11158) of chikungunya strains from adults (shown in closed circles) and children (shown in straight closed triangles). The tree was constructed using the neighbor-joining method with 1000 bootstrap resamplings and rooted with the E1 gene of the O'nyong-nyong virus. Reference sequences were obtained from the GenBank and EMBL databases; accession numbers are given in parentheses.

Comparative sequence analysis of chikungunya E1 gene from 15 CHIKV infected patients from KIMS revealed 98.3%–99.9% homology among them, clustering within the Central/East African genotype (Figure 3). Furthermore, all recently detected CHIKV strains worldwide including India clustered in this lineage with 93.4% to 99.9% homology. However, recently detected CHIKV strains from India appeared different from those of previous 1963 and 1973 outbreaks (Figure 3). Results were similar when the analysis was performed with amino acid sequences deduced from the E1 gene sequences (data not shown). Additionally we detected an amino acid change from lysine to glutamine at residue 132 in E1 gene among 5 of 8 CHIKV infected children, while none among the adults (data not shown). Of note, all these 15 cases were enrolled in the same place and during same period.

## Discussion

The present multi-centre study confirms and extends the findings of recent reports from India and other parts of world indicating a re-emergence of severe chikungunya disease which is becoming a major public threat in India [25], [27]. This is the first multi-centre study with sites from three distinct geographical locations except the eastern part of the country. The study was carried out with year round enrolment of subjects in comparison to previous CHIKV epidemiological data that was derived mainly from outbreaks [11], [12], [31]–[34]. For the first time both genomic (RT-PCR) and serology (IgM ELISA) based assays were employed throughout the study for laboratory confirmation of chikungunya in a large sample population. However our data on IgM antibody persistence emphasizes the need to analyze paired sample for IgM or IgG test for confirmation of chikungunya infection, particularly for those sampled after day-3 post illness when viremia is low.

Amongst the three centres, AIIMS and SMS had a relatively higher male∶female ratio. However this could be attributed to the social bias generally observed against females in the north and west India rather than to disease susceptibility based on gender. Various other studies conducted worldwide have shown inconsistencies on gender bias to disease susceptibility with most studies reporting each gender to be equally susceptible [31], [35], [36].

Discrepancies in the symptoms of Chikungunya among suspected cases across different studies warrant the requirement of a clear and well defined clinical profile of the disease. For example our study detected rashes more frequently among CHIKV cases as compared to other [12]. One reason for this discrepancy could be due to the study design. While earlier studies were retrospective focussing largely on outbreaks, our study was conducted throughout the year and across different sites.

Our study also demonstrated a link between chikungunya detection rate and geographical location of study centre. We observed highest percentage of cases at KIMS which is located along the coast in the southern part of the country compared to the other two centres that are landlocked regions with extended periods of dry summer and extreme winter. This is in agreement with findings of other studies those reported the prevalence of chikungunya mostly in the coastal regions including South India and South East Asia [4]–[7], [23], [24], [26], [27], [37], [38].

One interesting observation made in the present study was the near absence of chikungunya case at AIIMS site despite reports of dengue prevalence [39]. As both dengue and chikungunya are transmitted by the same vectors, it is not unreasonable to expect similar disease prevalence. However reports of the Delhi State Government showed large number of dengue cases in Delhi during 2008, while only few clinically suspected chikungunya cases were reported [40]. Similarly a study from Delhi detected CHIKV virus in serum samples of patients hospitalized during a dengue outbreak in the year 2006 [41]. Since then no confirmed cases of CHIKV infections have been reported from Delhi. One can speculate the reason for the absence of chikungunya to be simply a time factor and that in time CHIKV may make its way from the coastal regions to more inland areas such as Delhi. Hence, this warrants year round surveillance of CHIKV in regions that already have high prevalence of dengue. Beside the geographical location another reason for the lower detection of chikungunya could be due to silent infections that may have gone unnoticed in the past and thus may have primed the immune system against this pathogen. A study from Chennai, nearly a decade prior to the 1964 outbreak, observed high seropositivity among older subjects and they speculated the waning antibodies to CHIKV as a possible reason for the subsequent outbreak [42]. More studies need to be undertaken to prove this hypothesis and to further address the susceptibility of the Delhi populace to chikungunya infection. This is supported by reports of periodic outbreaks of dengue fever in Delhi every 3–4 years [39].

Chikungunya fever has been observed to occur mostly during the monsoon and post monsoon seasons during which time there is high breeding of both *Aedes albopictus* and *Aedes aegypti.* In India the first CHIKV outbreak in 1963 was observed during July to December, coinciding with the monsoon and post monsoon seasons. However, in the present study, CHIKV was detected throughout the year at KIMS centre although with higher rate during the monsoon season, a situation similar to dengue [39].

Our study has confirmed the findings of various other studies that certain symptoms like rashes, headache, joint pain/swelling and abdominal pain are significantly associated with chikungunya infections [12], [13], [31], [32].

Our finding on higher detection rate of chikungunya among adults as compared to children confirms most of those reported worldwide [11], [12], [31], [37]. In addition we found adults more frequently exhibiting symptoms such as rashes, headache, joint pain and joint swelling as compared to children [38]. Interestingly among rashes, erythrematous rash was frequently observed in adults while maculopapular rash was common in children confirming earlier reports in infants [43], [44]. Another interesting observation made in our study was the higher incidence of hepatomegaly among children aged below 5 years. Support for this result can be found in a recent pathogenesis study in mice that demonstrated hepatic involvement during chikungunya infection and showed CHIKV to replicate first in the liver before targeting the muscle and joints [45]. In addition, a recent review reported many atypical symptoms including raised levels of hepatic enzymes associated with chikungunya infection [25].

In addition to assessing clinical and epidemiological implications of chikungunya we tried to identify the molecular composition of the infecting strains. High nucleotide and amino acid homology was observed among the infecting strains which clustered within the Central/East African genotype confirming earlier reports that all recent CHIKV outbreaks in India have been a result of the Central/East African genotype [7], [37]. This is markedly different from the CHIKV strains that caused the outbreak in India during 1963–73 that resulted from the Asian genotype [46]. Our study also confirms earlier reports on the presence of A226V mutation in the E1 gene [24], [26], [34]. It has been suggested that a change in the amino acid at this particular position may provide a selective advantage for CHIKV transmission by mosquitoes by reducing their cholesterol dependence [6], [47]. Our study also observed a lysine-glutamine change at aa 132 in the E1 gene of CHIKV infecting children, an implication that warrants further investigation.

In conclusion, to our knowledge this is the first multi-centre study undertaken to determine the epidemiology of chikungunya fever across a wide age group with year round enrolment of large number of patients simultaneously in three different geographical regions of India. Both genomic and serology based assays were employed throughout the study for confirmation of CHIKV. Our study clearly demonstrates high incidence of CHIKV in India particularly in the southern region with moderate prevalence in the western and rare in the northern region. This warrants continuous CHIKV surveillance in India to determine the disease burden for improved healthcare.
